# Rational Design of Amino Acid-Modified Halide Perovskites for Highly Efficient and Cost-Effective Light-Emitting Diodes

**DOI:** 10.3390/ma18214982

**Published:** 2025-10-31

**Authors:** Hongyu Chen, Mingxia Qiu

**Affiliations:** College of New Materials and New Energies, Shenzhen Technology University, Shenzhen 518118, China; 202200301062@stumail.sztu.edu.cn

**Keywords:** FAPbBr_3_ quantum dots, amino acids, defect passivation, light-emitting diodes

## Abstract

Formamidinium lead bromide (FAPbBr_3_) quantum dots (QDs) have shown potential in light-emitting diodes (LEDs). However, their performance is constrained by surface defects and the limitations of charge transport. Zwitterionic ligands, owing to their twin functions of Lewis base coordination and electrostatic compensation, passivate surface defects of perovskite QDs. Some other zwitterionic ligands are high-cost, while amino acids, as zwitterionic ligands, are inexpensive, readily available, and have efficient passivation capabilities. Their short main chain and programmable side chain can control the volume and dipole at Å-scale range through functional group selection and feed ratio regulation, achieving interface energy level engineering. This work adopts green-emitting FAPbBr_3_ QDs as the model, tuning ligand properties by modifying side-chain functional groups, thereby achieving PLQY of 87.2%. Experimental results and DFT reveal that amino acids preferentially undergo coordination and can be further fine-tuned through conjugated contacts. Without severe site competition and without affecting coordination occupation and ligand uniformity, the EQE reaches 5.6% and the luminance exceeds 9000 cd/m^2^. This low-cost technology is easily scalable and broadly manufacturable, providing a replicable material and interface design route for green zone perovskite LEDs.

## 1. Introduction

Colloidal halide lead perovskite quantum dots (QDs) offer tremendous potential in light-emitting diodes (LEDs), memristors, solar cells, and photodetectors due to their variable bandgap, high absorption coefficient, and virtually unit photoluminescence quantum yield (PLQY). Shooshtari et al. demonstrated CMOS-compatible halide perovskite memristors. Especially Formamidinium lead bromide (FAPbBr_3_) QDs, as green-emitting materials, have gained substantial attention in display and lighting applications. However, one performance issue facing perovskite QDs is the stability degradation induced by surface defects, constrained by the limitations of traditional ligand charge transport [1,2,3,4,5,6,7,8,9]. Conventional strategies for enhancing QDs, such as ion doping and heterostructure/inorganic shell coating, typically rely on high-temperature and complex processes. In contrast, the room-temperature ligand coating strategy can be applied under mild conditions, offering a straightforward procedure with comparable efficiency. The perovskite crystal lattice is frequently passivated by long-chain organic ligands such as oleylamine (OLA) and oleic acid (OA) [10,11,12]. Nevertheless, these native long-chain ligands are primarily made of saturated alkyl chains, lacking π-conjugated or polarizable conductive functional groups. This constraint restricts exciton coupling and charge transport between nearby QDs [13,14].

Zwitterionic ligands, owing to their twin functionalities of Lewis base coordination and electrostatic compensation, display excellent efficacy in passivating surface defects of perovskite QDs [10,15]. Currently, various types of zwitterionic ligands have been employed to replace conventional alkyl ligands, including small-molecule sulfobetaine-based, natural and linear amino acids, zwitterionic phospholipids, macrocyclic/crown ether ligands, and other macrocyclic ligand structures. However, specific zwitterionic ligands, such as certain lipid derivatives and macrocyclic/crown ether derivatives, are relatively expensive. They often require unique synthesis, which includes complex techniques, significant costs, and poor storage stability [15,16,17,18,19,20,21].

Among numerous zwitterionic ligands, natural amino acids stand out as small-molecule zwitterionic ligands. They are very effective in defect passivation, enhanced conductivity, ease of acquisition, and low cost [22,23,24,25]. Specifically, amino acid molecules are short in size and abundant in coordination sites, which permits high coverage and multi-dentate passivation of quantum dot surfaces. Secondly, amino acids are widely available and affordable, so they meet the sustainability demands of large-scale production [15,26]. More crucially, amino acids exhibit short, extremely flexible leading chains and numerous side-chain configurations. By carefully selecting functional groups and adjusting the molar ratio of the ligand feedstock, the interactions between ligands and surfaces can be controlled. This permits selective passivation of defect locations and interfacial energy level engineering while preserving minimal conformational entropy penalties [27,28,29].

In this work, we utilize green-emitting FAPbBr_3_ QDs as a unified model. By using amino acid ligands within the Å-scale range, the volume and polarization can be carefully controlled, allowing the steric hindrance, dipole moment, and frontier orbital energy levels (HOMO/LUMO) to be shifted on the same framework. Compared with large molecule systems, amino acid ligands need to swap one side chain to drastically affect their volume and electrical characteristics. Moreover, their optical characteristics and conductivity can be progressively optimized by fine-tuning the structure. Based on this technique, this work gradually introduced four typical ligands, namely Alanine (Ala), Phenylalanine (Phe), Tryptophan (Trp), and Cysteine (Cys), and built up binary combination groups. Based on detailed characterisation and DFT simulations, a semi-quantitative assessment framework for the amino acid-perovskite interface was ultimately established, and the “first coordination, then conjugation” design approach was proposed appropriately. The results showed that, under the condition of no intense site competition and without disrupting the coordination occupation and uniformity of surface ligands, this strategy could have transferability within the device and process window of this study; in addition, this process is simple, the material cost is low, and it has certain promotion potential.

## 2. Materials and Methods

### 2.1. Synthesis Process of FAPbBr3 QDs

In the typical synthesis procedure, sequentially add 0.0781 g FAAc (0.75 mmol), 0.0759 g Pb(CH_3_COO)_2_·3H_2_O (0.2 mmol), 0.2090 g OAmBr (0.6 mmol), amino acids (with a 1:2 molar ratio of ligand to Pb^2+^), and 2 mL oleic acid, 8 mL n-octane. The four amino acids were 0.0090 g alanine (0.1 mmol), 0.0169 g phenylalanine (0.1 mmol), 0.0206 g tryptophan (0.1 mmol), and 0.0123 g cysteine (0.1 mmol), as well as mixed amino acids (0.0085 g phenylalanine (0.05 mmol) and 0.0103 g tryptophan (0.05 mmol), 0.0085 g phenylalanine (0.05 mmol) and 0.0062 g cysteine (0.05 mmol), 0.0103 g tryptophan (0.05 mmol) and 0.0062 g cysteine (0.05 mmol). The mixture was subjected to 7 min of 750 W high-power ultrasonication using a tip-mounted ultrasonicator under air conditions, while the reaction flask was placed in an ice-water bath for temperature control. The ultrasonication mode was set to a 3 s on/2 s off cycle. As the reaction progressed, the solution color changed from colorless to yellow-green, indicating the successful formation of FAPbBr_3_ QDs.

### 2.2. Purification

After the reaction is complete, centrifuge the crude product at 2000× *g* for 3 min to remove unreacted precursors, and then use the supernatant. Next, add ethyl acetate (30 mL, 3:1 volume ratio) to the supernatant. Distribute the mixture evenly into two centrifuge tubes and centrifuge at 9000× *g* for 5 min to collect the precipitate. Disperse each precipitate in 2 mL of n-hexane, then add 6 mL of ethyl acetate (3:1 volume ratio) to each. Centrifuge again at 9000× *g* for 5 min. The final precipitate was redispersed in 2 mL n-hexane and centrifuged at 2000× *g* for 3 min. The collected supernatant was to be the purified FAPbBr_3_ quantum dot solution, with a concentration of approximately 0.0002 mol/4 mL.

### 2.3. Fabricating Process of PeLED Device

Device fabrication utilized patterned ITO conductive glass as the bottom electrode, which was sequentially ultrasonically cleaned in deionized water, acetone, and isopropanol before undergoing 20 min of ozone treatment. The PEDOT: PSS solution was filtered through a 0.45 μm membrane, spin-coated at 4000 rpm for 40 s, and annealed on a 150 °C hot plate for 15 min to form the hole injection layer. Subsequently, a 4 mg/mL poly-TPD chlorobenzene solution was spin-coated at 4000 rpm for 60 s and annealed at 150 °C for 20 min to form the hole transport layer. The emissive layer was formed by spin-coating a purified FAPbBr_3_ quantum dot solution (2000 rpm, 60 s) and annealing at 60 °C for 2 min. This process was repeated twice to enhance film thickness and uniformity. Finally, under high vacuum (<4 × 10^−4^ Pa), the device structure was completed by sequentially thermally evaporating the electron transport layer TPBi (40 nm), LiF (1 nm), and cathode Al (100 nm).

## 3. Results

### 3.1. Optical Properties of Amino Acid Ligand-Doped Materials

**Figure 1:**
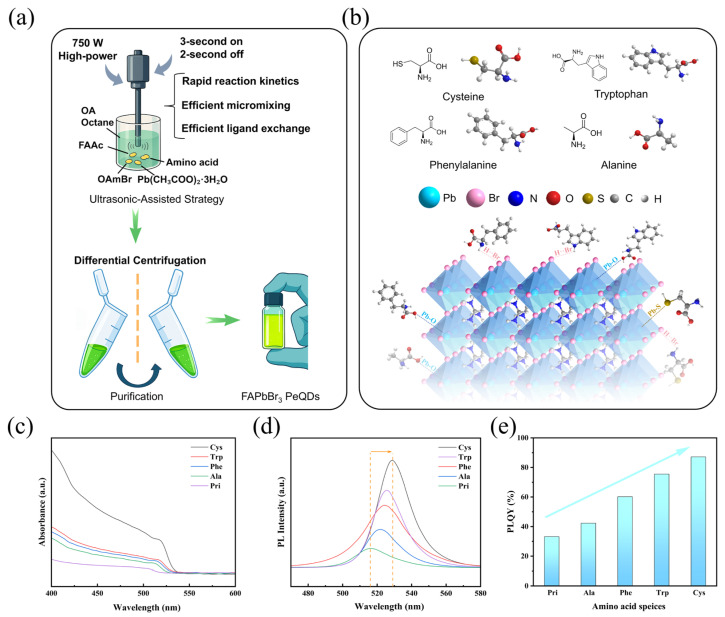
(**a**) Schematic illustration of the ultrasonic-assisted strategy for preparing FAPbBr_3_ perovskite quantum dots (PeQDs). (**b**) Schematic diagrams of the configurations of four amino acids acting on the surface of the Pristine (Pri) FAPbBr_3_ PeQDs sample. (**c**) UV-vis absorption spectra of the FAPbBr_3_ samples. (**d**) PL emission spectra of the FAPbBr_3_ samples. (**e**) Comparison of photoluminescence quantum yields (PLQY) for the FAPbBr_3_ QDs.

The schematic diagram of the ultrasonic-assisted synthesis process adopted in this work (Figure 1a): Amino acid small molecules are mixed with precursor powder, reacted under 750 W high-power ultrasonic action, and then purified by multi-stage centrifugation to obtain uniform and green-emitting FAPbBr_3_ QDs; Figure 1b shows the chemical structures of four representative amino acids and their interactions with the surface of QDs through coordination or hydrogen bonds. The FAPbBr_3_ QDs modified with four distinct amino acid ligands, namely Alanine (Ala), Phenylalanine (Phe), Tryptophan (Trp), and Cysteine (Cys), all displayed outstanding stability. They presented vivid green emissions in the visible light region (with emission peaks approximately at 515–530 nm) [2,26]. Due to the differences in volume and polarization of the side chains of R-based programmable amino acids, the regulation of the interface state of QDs is dramatically different, which leads to systematic changes in their spectral properties and photoluminescence quantum yields (PLQYs).

Figure 1c displays the ultraviolet-visible absorption spectra of FAPbBr3 QDs modified with different amino acid ligands. All five QDs displayed distinct absorption initiation edges in the range of 515–525 nm, and the primary absorption sources emanated from the band edge transitions of perovskite crystals within the quantum confinement. The initial absorption edge of the Pri-QDs exhibits the bluest shift, indicating that their particle size is the smallest and the confinement effect is the strongest. However, its overall absorption intensity is the lowest, presumably due to its low concentration or poor dispersion, and more surface imperfections, which lead to a fall in light absorption efficiency. In contrast, Cys-QDs displayed the most vigorous absorption intensity and the most redshifted absorption edge, suggesting that their particle size was quite large. However, their surfaces were generally passivated, the dispersion is better, and the absorption cross-section is more substantial, which may be due to the superior coordination stability and homogeneity of Cys [23,30]. The absorption edges of Trp-QDs, Phe-QDs, and Ala-QDs sit between the two, displaying a minor long-wave shift; their regulatory influence on particle size is not as straightforward as that of Cys-QDs. Figure 1d displays the photoluminescence (PL) emission spectra of the QDs. All QDs displayed green edge-banded luminescence between 515 and 530 nm, and the intensity variation trend was comparable with the UV-VIS data. Similar studies have shown that FAPbBr_3_ QDs also exhibit narrow FWHM and high PLQY at approximately 520 nm. The Cys-QDs exhibit the most vigorous luminescence intensity and the narrowest half-height width (FWHM), indicating high exciton radiation recombination efficiency and few trap states [2,30]. In contrast, the luminescence intensity of the Ala-QDs was the weakest, and the spectral line broadening was apparent, suggesting that there was a high number of unpassivated defect sites on its surface, and traps aided recombination occurred. Previous investigations have pointed out that weakly coordinating ligands are complex to passivate Pb^2+^/Br^−^ vacancies, hence generating non-radiative recombination and resulting peak line broadening [10]. The luminescence intensities of Trp-QDS and Phe-QDS are modest, with Trp-QDS marginally superior to Phe-QDS. Its enhanced performance may be attributed to the π interaction of the indole structure on the surface or to more hydrogen bonds [25]. The PLQY comparison of each sample is given in Figure 1e. The PLQY of the Pri-QDs was 33.2%. After treatment with the Alanine ligand, it increased to approximately 42.3%. Further modification with Phenylalanine and Tryptophan improved the PLQY to approximately 60.2% and 75.5%, respectively. Among them, the Cys-QDs achieved the highest PLQY (about 87.2%), indicating that it has the most substantial passivation impact on surface defects [23]. The trend of PLQY clearly displays the influence of diverse ligand side chain functional groups on the radiative recombination efficiency of PeQDs. Specifically, this means Cys > Trp > Phe > Ala > Pri, but experimental characterizations and DFT calculations may explain this trend.

By merging these three sets of images, it can be observed that the optical characteristics of QDs (UV-VIS, PL, PLQY) show a stepwise shift in the same pattern (Cys > Trp > Phe > Ala > Pri) with R-group fine-tuning. Factors such as the polarity of side chain groups, electron supply capacity, and spatial volume have a significant impact on the passivation effect of surface defects of PeQDs. Therefore, based on the stepwise changes in its optical performance, we subsequently selected the Cys-QDs with the best performance and the Ala-QDs with the worst performance, and conducted in-depth comparative analysis with the Pri-QDs to further reveal the relationship between the ligand chemical structure and the regulation of the surface state of QDs.

### 3.2. Research on the Topological Structure of Amino Acid Ligand Doping

**Figure 2:**
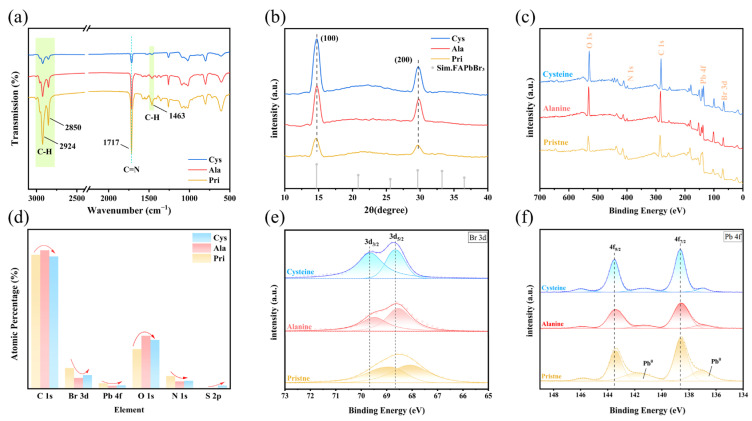
(**a**) FT-IR spectra of Pri-QDs, Ala-QDs and Cys-QDs, (**b**) X-ray diffraction (XRD) patterns, (**c**) full X-ray photoelectron spectroscopy (XPS) spectra, (**d**) atomic percentages of elements calculated from the whole spectra, (**e**) high-resolution XPS spectra of Br 3d and (**f**) high-resolution XPS spectra of Pb 4f.

The essence of the optical response originates from the evolution of crystal and interface structures. So we introduced Ala and Cys, respectively, as ligands in order to conduct Fourier transform infrared (FTIR) spectroscopy analysis (Figure 2a). We discovered that the C-H stretching peaks (2924, 2850, and 1463 cm^−1^) of the Ala-QDs and Cys-QDs all dropped, indicating that short-chain amino acids replaced some long-chain OA/OLA [31,32]. In the FT-IR spectra of FAPbBr3 QDs, the 1717 cm^−1^ peak belongs to the C=N stretching vibration of the FA^+^ cation. After directly adding amino acids into the precursor solution, this absorption peak dramatically reduced and showed no red shift. The attenuation amplitude of Cys-QDs was larger than that of Ala. This phenomenon is connected in coordination ability brought about by the structural fine-tuning of the side chain: Alanine only provides -NH_2_/-COOH, with limited metal affinity; Cysteine additionally introduces a soft Lewis base -SH group, which preferentially coordinates with Pb^2+^ during the early nucleation stage and more strongly hinders the ordered insertion of FA^+^, resulting in a more pronounced weakening of the C=N signal. At the same time, no apparent SH vibration peak was identified near 2570 cm^−1^, indicating that most of the mercapto groups have participated in coordination complexation rather than remaining in a free form. The results above implicitly show that, at the spectral level, by adding stronger electron-donating functional groups (-SH) through side chain fine-tuning, the amino acid can significantly strengthen the control ability of the amino acid on the lattice composition and surface defects of QDs [33].

To evaluate the influence of amino acid insertion on the crystal structure of QDs and the difference trend compared to amino acids, we compared their X-ray diffraction (XRD) patterns (Figure 2b). The results showed that both the Pri-QDs and the modified version of QDs presented a cubic perovskite phase (space group Pm3m). Still, compared to the Pri-QDs, the prominent diffraction peaks of the Ala-QDs and Cys-QDs (such as the (100) peak at 14.54° and the (200) peak at 29.64°) all shifted approximately 0.13° to the right, indicating a slight reduction in the lattice spacing and a slight lattice contraction [2]. As demonstrated by FTIR, the inclusion of amino acids into precursor modifications may trigger the loss of FA^+^ cations, lowering FA^+^ occupancy and generating modest lattice contraction. This emerges as a rightward shift in the primary diffraction peak in XRD. Liuquan et al. also noticed that the chemical characteristics of ligands and their dynamic behavior within the reaction system strongly influence the stability of organic cations and crystal formations [34]. What is more remarkable is that we noticed that the diffraction peak intensity of the Cys-QDs was substantially higher than that of the Ala-QDs. This would indicate that it has a stronger directing influence on crystal development, boosting the crystal orientation or crystallinity, which is directly related to its stronger coordination capacity of the -SH group.

The XPS full spectrum and atomic percentage analysis (Figure 2c,d) show that after ala treatment, the total proportion of C 1s and O 1s rose to approximately 90%. In comparison, Pb 4f and Br 3d decreased sharply to 1.35% and 5.10%, respectively, indicating that the -COO^−^/-NH_3_^+^ framework corresponds to a higher surface organic coverage. In comparison, the cysteine sample still retained 1.58% Pb 4f and 6.38% Br 3d, and added 1.46% S 2p signal, demonstrating that -SH can form site coordination with uncoordinated Pb^2+^, resulting in comparatively lesser coverage of the interfacial layer. These results indicate that the careful adjustment of the Å-scale R-group has a certain impact on the density and coordination preference of the surface coating of FAPbBr_3_ QDs.

We conducted XPS investigation of Br 3d on QDs, as shown in Figure 2e. In the Pri-QDs, the Br 3d_5/2_ and Br 3d_3/2_ peaks were situated at around 68.1 eV and 69.2 eV, respectively, demonstrating typical spin–orbit splitting characteristics. After the injection of alanine, the two peaks significantly blue-shifted. The hydrophobic -CH_3_ side chain of alanine had a modest influence on the electronic structure around Br, making it challenging to coordinate with the perovskite surface or passivate defects effectively, and the binding energy shift was not considerable. In contrast, the Br 3d doublet in the Cys-QDs showed a more pronounced blue shift, going up to roughly 68.6 eV and 69.7 eV, respectively, indicating a decrease in the electron cloud density of the Br in its environment. This might be owing to the strong coordination interaction created between the -SH group in the Cysteine molecule and the Pb^2+^ ion, thereby changing the local electronic structure of Br [32]. At the same time, the linewidth of the Br 3d peak in the Cys-QDs was narrower compared to the Pri-QDs and the Ala-QDs, and the splitting was more apparent. Similar behaviors were also seen in the bound N-dodecylbenzene sulfonic acid (DBSA)-modified CsPbBr_3_ nanocrystal system [35].

Further Pb 4f XPS experiments were undertaken on the Pri-QDs, the Ala-QDs, and the Cys-QDs (as shown in Figure 2f). In the Pri-QDs, the Pb 4f_7/2_ central peak was placed at around 138.5 eV, and the 4f_5/2_ central peak was at roughly 143.3 eV. In the Cys-QDs, the Pb 4f_7/2_ peak significantly shifted to approximately 138.6 eV, and the 4f_5/2_ central peak rose to approximately 143.5 eV; while in the Ala-QDs, the peak position also slightly increased, but the change was not significant compared to the Pri-QDs. For Cys-QDs, the Pb 4f_7/2_ and 4f_5/2_ peaks show a particular blue shift, which is mainly owing to the strong coordination capacity of the -SH group in the Cys molecule. The -SH group is a soft base and can form a strong Pb-S coordination bond with Pb^2+^, a soft acid [10]. In contrast, alanine only has an inert -CH_3_ side chain. Although its carboxylic group has some coordination capacity, it lacks the soft base functional groups necessary to establish a strong coordination bond with Pb^2+^ [22]. Three low-intensity components are observed on both sides of the prominent peaks of Pb 4f_7_/_2_ and 4f_5_/_2_ in the Pri-QDs: a Pb^0^ double peak at approximately 137 eV and 142 eV, and a high binding energy Pb tail peak at 145–147 eV [36,37,38]. Following the introduction of alanine, the double-peak integral area for Pb^0^ reduced dramatically, showing that the monodentate carboxyl group effectively prevents the production or exposure of metallic lead by coordination with Pb^2+^. Meanwhile, the high-energy tail peak remained essentially constant. When further modified with cysteine, the Pb^0^ signal at 137 eV was further weakened, and the shoulder peak at 142 eV was significantly enhanced compared to the Ala-QDs; combined with the synchronous S 2p_3/2_ signal, this shoulder peak can be attributed to Pb-S coordination, confirming that the -SH/-COOH dual-site synergy further passivated the residual Pb^0^. The systematic evolution of the shoulder peak reveals that alanine reduces lead defects through single-point coordination with the carboxyl group. At the same time, cysteine profoundly repairs defects via combined Pb-S and Pb-O coordination, exhibiting superior regulatory effects in electronic structure reconstruction.

Figures S1–S3 indicate that only Cys-QDs exhibit an S 2p doublet assignable to thiolate-like Pb-S coordination, with no free -SH/S-S features; the concomitant disappearance of the -SH stretching band in FTIR further supports this [10,31]. O 1s analyses show that, relative to Pri-QDs, Alanine introduces carboxylate Pb-O coordination and cysteine strengthens it, evidenced by the emergence/intensification of a lower-binding-energy shoulder [2,39]. In N 1s, amino-acid modification slightly shifts the FA-related component and yields a higher-energy -NH_3_^+^ feature from the ligands, consistent with mild ligand-lattice interactions during nucleation/growth [31,32,33].

The FT-IR/XRD/XPS results indicate that there is a specific structural-optical coupling in the system: the emission difference mainly results from the changes in the interface structure and surface coordination chemistry of the crystals caused by the ligands; however, the single-ligand control also shows substantial differences. To achieve fine control for devices, the following will undertake research on binary ligand combinations, combine DFT to quantitatively evaluate amino acid small molecules, and verify with indicators such as EQE of PeLED.

### 3.3. Multiligand Studies and Electronic Structure Research

Fine-tuning based on the R base structure improved the optical/topological structure of FAPbBr3 QDs. We further understand the structure-optical coupling through amino acid combinations. Considering the final optical performance for PeLED, this work prioritized picking the three amino acids (Cys, Phe, Trp) with the best PLQY, then combined them in equal molar ratios in pairs to evaluate whether there was a synergistic enhancing impact.

**Figure 3:**
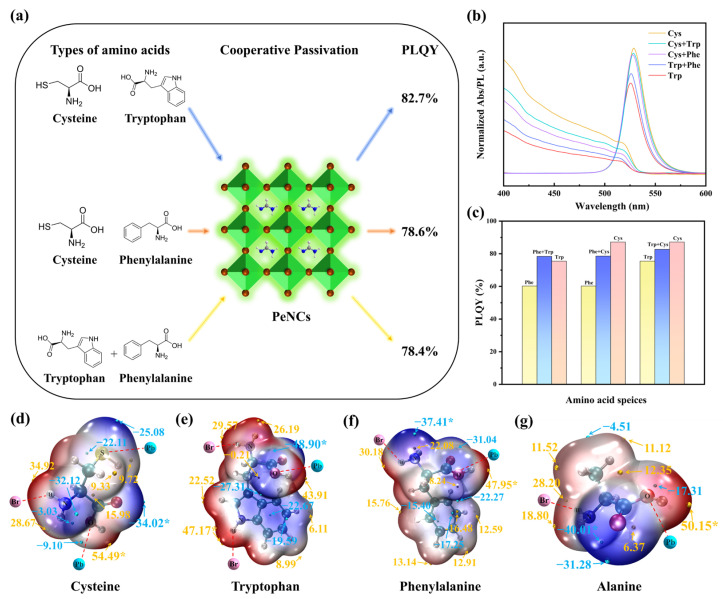
(**a**) Schematic illustration of synergistic interactions between paired amino acids on the perovskite surface. (**b**) Normalized absorption and photoluminescence (PL) spectra of mixed amino acid systems with tryptophan and cysteine are used as references. (**c**) Comparison of PLQY for perovskite quantum dots treated with single and dual amino acid combinations. (**d**–**g**) Electrostatic potential (ESP) maps for the four amino acid molecules, * is a maximum value point. The red line represents the interaction with the surface of the perovskite.

Figure 3b depicts the normalized absorption and PL emission spectra of the binary ligand system, with the Cys-QDs and Trp-QDs serving as references. As the ligands change from Cys, Cys + Trp, Cys + Phe, Trp + Phe, and Trp in series, the absorption edge and PL peak location shift towards the blue. The spectral positions of Cys + Trp and Cys + Phe are near those of the Cys-QDs, further demonstrating that Cys dominates in the complex system; however, the peak position of the Trp + Phe QDs is somewhat displaced towards the longer wavelength compared to the Trp-QDs.

Figure 3c indicates that the PLQY values of the three binary ligand systems are all higher than those of their respective weaker single-ligand systems. Fascinating is the Phe + Trp combination, with a PLQY of up to 78.4%, slightly above Trp (75.5%) and Phe (60.2%), suggesting a nonlinear synergistic enhancing impact. In contrast, Phe + Cys (78.6%) and Trp + Cys (82.7%) also boosted PLQY, but remained lower than the 87.2% of the Cys-QDs, demonstrating that Cys dominates the coordination process in these two groups. This occurrence is consistent with the high coordination affinity of the Cys for the exposed Pb^2+^ sites. Phe or Trp principally support the surface shell layer by providing steric hindrance and hydrophobic barriers, playing an auxiliary role. Overall, the order of the core wavelength of the spectrum is perfectly compatible with the PLQY values.

Furthermore, in the ESP mapping, the extreme value distribution of each amino acid molecule clearly represents the charge distribution properties of its functional groups, which is related to the observed sequence (Cys > Trp > Phe > Ala > Pri). For Cys, its ESP map displays a specific deep blue band surrounding the sulfur atom, indicating a high negative electrostatic potential. This very negative area corresponds to the sulfur atom’s lone pair of electrons, suggesting that it is a classic Lewis base site with strong nucleophilic characteristics. Therefore, the sulfhydryl group (-SH) in the Cys molecule is deemed capable of creating stable coordination bonds with uncoordinated Pb^2+^ on the perovskite surface. In contrast, the minima of the negative ESP for Trp and Phe are primarily distributed near the carboxyl oxygen atom and the amino nitrogen atom. Although strong negative potential sites exist in other areas (such as the nitrogen on the indole ring), efficient coordination is difficult to establish due to severe steric hindrance. Ala similarly exhibits a negative ESP minimum at the amino and carboxyl groups. Still, it has a limited number of functional groups and fewer Lewis base sites that can engage in coordination. For Cys, the positive ESP is predominantly concentrated at the carboxyl hydrogen, exhibiting a bright red region, indicating that there is a partial positive charge at this site, and it is prone to experience electrostatic attraction with anions. In addition to the carboxyl hydrogen, the N-H group on the indole ring of Trp also generates a positive electrostatic hotspot, which can act as a hydrogen bond donor to produce N-H···X^−^ hydrogen bonds with halide ions. Therefore, the indole N-H in the Trp molecule, as well as the -NH_3_^+^ in Phe and Ala, may have electrostatic attraction or hydrogen bond interactions with the uncoordinated Br^−^ or halide vacancies on the surface, therefore passivating these negative charge defects [34].

To further investigate the balance of the charge distribution, the number and spatial distribution of the ESP extreme points can be employed as a measure. When a molecule contains several positive and negative ESP extreme points, it shows a multi-center characteristic; conversely, if the extreme points are concentrated, it is more comparable to a single-pole dipole characteristic. In the ESP diagram of Cys, there is a negative value near the carboxyl oxygen, with secondary negative values around the sulfur atom, and positive values near the amine and carboxyl hydrogens. This multi-valued distribution permits multi-point anchoring at the interface [24,40,41]. For Trp, the carboxyl oxygen is the main negative point, with secondary negatives above and below the indole π system; the amino group and the indole N-H form two positive points. The indole ring can undergo cation-π-π interaction with Pb^2+^, while the indole N-H forms hydrogen bonds with Br^−^. In contrast, Phe and Ala show more concentrated extrema. Phe has a negative value at the carboxyl oxygen and a positive value at the amino group, while the benzene ring provides only a weak cation-π-π interaction with Pb^2+^ [25]. The overall charge distribution can be modeled as a single dipole along the main chain; when adsorbed on the perovskite surface, it largely contributes through the ends, and the central hydrophobic region has virtually no electrical influence. Ala is simpler, with extrema localized at the carboxyl and amino ends, corresponding to a typical dipole and lacking multi-point cooperative passivation [42].

The quantity, intensity, and practical action of ESP extreme points together govern the charge balance and interface interaction mode of molecules. Polymodal molecules such as Cys and Trp have more dispersed internal charges. They can anchor at multiple sites on the perovskite surface, which is helpful for simultaneously passivating Pb^2+^ and halogen-related defects, and avoiding a strong dipole that causes a drastic curvature of the interface energy level. In contrast, molecules with relatively limited effective strong extreme points, such as Ala and Phe, exhibit stronger single dipole characteristics, which may have a better local passivation effect on a specific type of defect (such as Pb^2+^); however, the overall coordination is weaker. Thus, these results provide a possible explanation for the observed sequence (Cys > Trp > Phe > Ala > Pri) [39,40,43].

**Figure 4:**
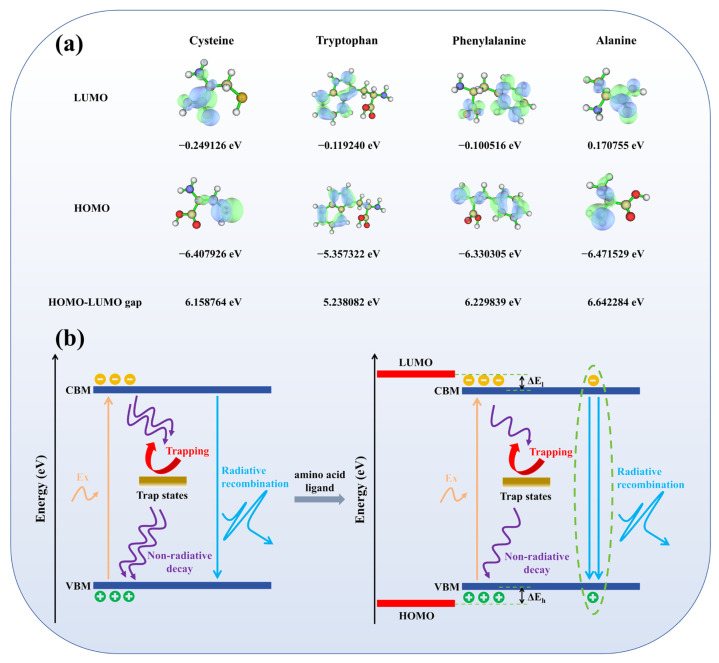
(**a**) Frontal molecular orbital distribution and energy level diagram of four amino acids, along with corresponding energy level values and band gaps, and (**b**) Mechanism diagram of FAPbBr_3_ quantum dots without modification and mechanism diagram after amino acid modification.

HOMO/LUMO can be used to analyze the energy level alignment with the band edges (VBM/CBM) of FAPbBr_3_ and the orbital spatial coupling at the interface, the DFT results reveal the HOMO and LUMO energy levels and their energy gaps for each ligand: for example, Trp (indole) shows a higher HOMO and a smaller gap, whereas Ala shows a lower HOMO and a larger gap. In terms of energy level alignment, the CBM of FAPbBr_3_ is roughly −3.4 eV, while the VBM is approximately −5.6 eV [44]. The LUMO of the four amino acid ligands in this study ranged from −0.25 to +0.17 eV, all of which were substantially higher than the CBM; their HOMO was close to or below the VBM. This means that, except for Trp, the rest of the interfaces are basically in type-I alignment (Figure 4b). Trp is closer to the band edge on the valence band side and corresponds to “near-band-edge alignment” [45]. In this sample and processing conditions, its alteration still effectively suppresses the non-radiative channel and retains strong luminescence. Under this premise, the key to determining the amplitude of the luminescence enhancement is the relative energy level proximity of the frontier orbitals to the band edge, and whether the spatial distribution of LUMO/HOMO covers the anchoring sites and forms an effective orbital docking with the unsaturated surface sites of Pb-Br [10].

Under this framework, the performances of different ligands reveal unique variances. The LUMO of Cys is the lowest (−0.249 eV), and the unoccupied state electron density intensely concentrates towards the quantum dot binding area. While preserving the I-type potential barrier, the interface can form effective exciton coupling, and the related PLQY is maximized. Thanks to indole conjugation, the HOMO of Trp is elevated to roughly −5.36 eV; compared with the VBM of FAPbBr3 (−5.60 eV), it shows a 0.24 eV shift. The front-line orbitals are predominantly on the indole ring, and there is still considerable electron density around anchor sites such as -COO^−^/-NH_2_, which is conducive to coordination and decreases non-radiative channels. Therefore, in defect-controlled QDs, Trp still offers a moderate luminosity gain [45,46,47]. Although the LUMO of Phe is slightly distributed in the -COOH region, it is mainly confined to the benzene ring, lacking directionality towards anchor sites; the overlap with the Pb^2+^ neighborhood is limited, resulting in weak interface coupling and limited PLQY gain. Even so, π conjugation and a hydrophobic shell provide certain surface shielding, making its overall performance better than that of Ala. Ala, due to its basic structure and non-conjugation, has LUMO almost confined to -COOH, with poor spatial expansion/overlap, missing effective orbital bridging, and thus the weakest luminescence enhancement. When the ligand HOMO is close to the VBM and the frontier orbitals (especially LUMO) cover anchor sites spatially—and both match with the I-type barrier—the interface energy dissipation is minimized. Thus, the spatial docking of HOMO/LUMO becomes a key parameter governing energy-coupling efficiency and the eventual PLQY enhancement [45,46,47].

In conclusion, the regulation of FAPbBr_3_ QDs by amino acid ligands is mainly achieved through the adjustment of the perovskite colloid interface. Specifically, it primarily comes from two paths: one is the coordination between functional groups and unsaturated ions, directly reducing surface defect states; the other is the conjugation of side chains through electron repositioning and short-range coupling, further enhancing luminescence. In this system, the priority and efficiency of coordination passivation surpass those of conjugation. If defects are not reduced, simply adding conjugation often fails to yield a real PLQY improvement.

In binary ligands, the PLQY is higher than that of their corresponding monoligands. The PLQY of Trp + Phe reaches 78.4%, exhibiting nonlinear synergy. This may arise from conjugation-coordination synergy under conditions without substantial site rivalry, where Trp provides π conjugation and N-H···X^−^ coordination, and Phe provides aromatic shielding and short-range coupling. Neither significantly competes for the same sites, enabling more uniform coverage and a more continuous interface; when defects are suppressed, the gain of exciton coupling improves light-emission efficiency [48,49,50]. In contrast, Cys + Trp and Cys + Phe also increase PLQY (82.7% and 78.6%) but remain below Cys monoligand (87.2%): the -SH/-COO^−^ groups in Cys have high affinity for Pb^2+^, preferentially occupying and saturating key sites so that Cys dominate defects and spectral position. Adding Trp or Phe tends to compete at the same sites and mainly strengthens the hydrophobic barrier and shell stability, with limited gains in energy-level alignment and orbital coupling; thus, it is difficult to exceed the Cys monoligand. Overall, for amino-acid tuning at the perovskite interface, coordination dominates while conjugation is secondary; only when site competition is weak and coordination has minimized defects can mild conjugation provide a further (limited) increase in light-emission efficiency.

### 3.4. Perovskite Light-Emitting Diodes

**Figure 5:**
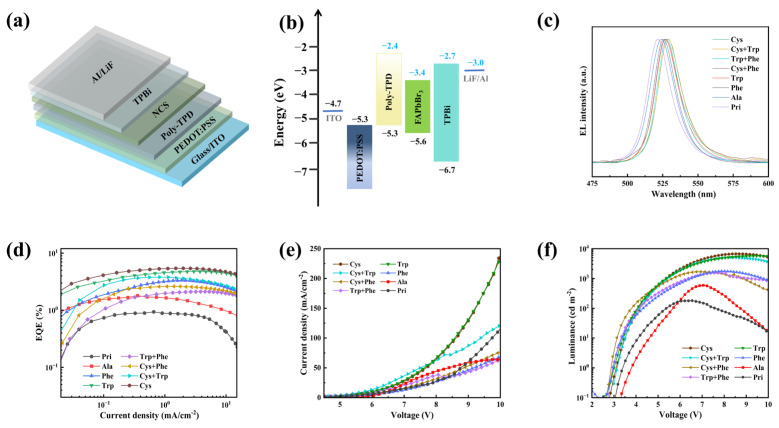
(**a**) Schematic diagram of the FAPbBr3 PeLED device structure. (**b**) Energy level alignment diagram of each functional layer in the device structure. (**c**) Electroluminescence (EL) spectra of devices with different ligand treatments. (**d**) Relationship between the external quantum efficiency (EQE) and current density of the devices, (**e**) current density-voltage characteristics, and (**f**) relationship between luminance and voltage.

To verify the effectiveness of small molecule ligands in enhancing luminescence by starting from structural fine-tuning, we used amino acid small molecules modified by the R group and binary ligand-modified FAPbBr_3_ QDs as the emission layer to construct a PeLED with a typical structure. The device structure is ITO/PEDOT: PSS/Poly-TPD/FAPbBr3QDs/TPBi/LiF/Al, as shown in Figure 5a. Here, PEDOT: PSS serves as the hole injection layer, and Poly-TPD (approximately 20 nm) serves as the hole transport layer. The quantum dot emission layer was spin-coated in a glove box and then consecutively deposited in a vacuum thermal evaporation system with TPBi (40 nm), LiF (1 nm), and Al (100 nm), which serve as the electron transport layer, electron injection layer, and top electrode, respectively.

Figure 5b displays the energy level structure of this device. Holes are injected from ITO (−4.7 eV) successively into PEDOT: PSS (−5.3 eV) and Poly-TPD (−5.3 eV) HOMO, and then enter the emission layer (FAPbBr_3_, VBM approximately −5.6 eV); while electrons are injected from Al (−3.0 eV) into LiF, and then pass through TPBi (LUMO approximately −2.7 eV) to enter the conduction band of the quantum dots (CBM approximately−3.4 eV). The LUMO of Poly-TPD (−2.4 eV) is higher than that of FAPbBr3, which serves to limit reverse electron diffusion; whereas the deep HOMO of TPBi (−6.7 eV) effectively blocks the escape of holes [44,51].

PL reflects photon-driven radiative recombination, while EL arises from electrically driven recombination after carrier injection. The EL peak position/half-width and EQE may deviate from PL. Compared with PL, all ligand-modified QDs show a slight EL red shift (Figure 5c), whose magnitude correlates with the initial PL hue: Trp/Ala (slightly blue in PL) red-shifts more, whereas Cys/Cys + Trp (slightly red in PL) changes little. Ultimately, EL peaks converge to 525–530 nm. Prior studies attribute the EL-PL red shift to combined effects: shallow-trap/non-ideal band-edge recombination, the quantum-confined Stark effect, and energy/carrier redistribution (e.g., FRET) toward lower-energy or larger QDs [52,53,54].

Figure 5d demonstrates that the Cys-modified device achieves a peak external quantum efficiency (EQE) of nearly 5.6%. Trp and Cys + Trp have efficiencies of 4.8% and 3.8%, while the other binary ligands sit between Ala and Phe. The unmodified QD EQE is less than 1%. The sulfur-containing ligand Cys, through -SH/-COOH, forms a dual-site coordination with the unsaturated Pb^2+^, which can reduce trap states and promote radiative recombination without significantly increasing the tunneling barrier. In contrast, π-conjugated aromatic molecules (Trp, Phe) may impair passivation and induce injection imbalance due to steric hindrance and unequal dipole distribution. The performance of electroluminescence (EL) is dictated by continuous carrier injection and interface energy level alignment, rather than by the rise in radiative probability. Based on this mechanism, only when the coordination sites are occupied and uniform coverage, coherent dipole orientation, and energy level alignment can the PLQY in the solution state be translated into EQE gain [55,56,57]. The single ligand of Cys, Trp, or Phe is more likely to generate uniform coverage, coherent dipole orientation, and energy level alignment. It achieves a greater EQE than a binary system. EL has resolution for interface electronic transport, and the synergy of binary ligands is only possible under the condition of continuous carrier injection and interface energy level alignment.

The J-V characteristic (Figure 5e) supports this explanation. The device with a single Cys and Trp ligand displayed a greater current density at the same voltage, indicating better carrier injection and transport capabilities. The reduced current density of the binary system of Cys + Phe and the early entry into the injection-limited range show that the coverage uniformity and dipole orientation still need to be adjusted. Studies indicate that if a vertical component gradient is formed during the film process, it will diminish the local effective conductance and cause spatial charge buildup, hence magnifying non-radiative channels. The L-V curve (Figure 5f) results are consistent with the above trend. The maximum brightness of the Cys and Trp-modified devices both exceeded 9000 cd/m^2^, and high brightness output could be achieved at low voltages while maintaining a good voltage linear relationship, indicating good synergy in carrier injection, exciton recombination, and light extraction. However, the Ala and Pri-modified devices showed a brightness peak at 7–8 V, and then decreased, indicating a propensity to efficiency roll-off under high injection density, possibly due to incomplete surface passivation leading to an increase in trap state density, thereby enhancing Auger recombination and other non-radiative channels [52,53,54].

Under the same device structure and process conditions, the EQE of PeLED is regulated by the continuity of carrier injection/transmission and the alignment of interface energy levels. The Cys monoligand can form a dense and low-barrier interface due to its strong coordination with Pb^2+^, achieving the highest EQE. This binary ligand failed to convert the advantage of solution-state PLQY into EQE gain because the coverage and energy level alignment did not form sufficient synergy. In this work, natural amino acids were employed to achieve micro-tuning of the R group at room temperature in solution. As shown in Table S1, compared with popular synthetic zwitterionic ligands (such as phosphocholine, sulfobetaine derivatives), amino acids provide both structural variety and significant cost advantages. The cost of a single dose is nearly two orders of magnitude cheaper (0.083–0.399 US dollars, purity ≥ 98%). Based on the device performance and spectroscopic evidence, we propose a design principle: first, use coordination small molecules to establish a low-trap interface base, and then introduce a moderate conjugated ligand for fine-tuning without destroying the coverage and energy level alignment, without causing intense competition at the site. It has the advantages of low cost, room-temperature realization, and easy amplification, which have direct reference value for material screening and large-scale manufacture of PeLEDs.

## 4. Conclusions

Perovskite shows considerable potential for LED. However, its performance is hindered by the stability decline, surface defects, and the limited charge transport of conventional ligands. Amino acids, as naturally occurring zwitterionic ligands, stand out for effective defect passivation, increased conductivity, easy availability, and low cost. They are regarded as an option to replace long-chain alkyl ligands. In this work, using green-emitting FAPbBr_3_ quantum dots as a unified model, amino acid ligands are used to achieve volume and polarization fine-tuning within the Å-scale range, so that steric hindrance, dipole moment, and HOMO/LUMO change on the same framework, thereby achieving an optical performance of 87.2%. At the same time, XPS studies and DFT calculations demonstrate that amino acid small molecules favor coordination and may further be fine-tuned through conjugated contacts. Finally, by screening the amino acid modifier components, improved band-edge docking is accomplished, and the EQE is further raised to 5.6% and the luminance exceeds 9000 cd/m^2^. This technique relies on common amino acid raw materials and solution methods, has minimal cost ($), and has the feasibility of easy amplification and promotion for manufacture, giving a replicable material and interface design route for green perovskite LED.

## Data Availability

The original contributions presented in this study are included in the article/Supplementary Material. Further inquiries can be directed to the corresponding author.

## Supplementary Materials

The following supporting information can be downloaded at: https://www.mdpi.com/article/10.3390/ma18214982/s1.
