# Supplementary material for: Rational Design of Amino Acid-Modified Halide Perovskites for Highly Efficient and Cost-Effective Light-Emitting Diodes
Source: Materials (Basel). 2025 Oct 31;18(21):4982. doi: 10.3390/ma18214982 (PMC12608446; doi:10.3390/ma18214982)
Supplement: Supplementary file 1 [file materials-18-04982-s001.zip › materials-3945156-supplementary.pdf]

Supporting information

Rational Design of Amino Acid-Modified Halide Perovskites for Highly Efficient and Cost-Effective Light-Emitting Diodes

Hongyu Chen[1], Mingxia Qiu[1*]

[1]College of New Materials and New Energies, Shenzhen Technology University, Shenzhen 518118, China

Corresponding Author

[*]E-mail: qiumingxia@sztu.edu.cn

Page number: Page 1-8

Partial experimental section

Figure number: Figure S1 S2 S3

Table number: Table S1

References

Materials. Formamidinium acetate (FAAc, 99%; MREDA, Beijing, China), lead(II) acetate trihydrate $Pb(CH_3COO)_2·3H_2O$, 99.999%; TCI (Shanghai) Development Co., Ltd., Shanghai, China), oleylammonium bromide (OAmBr; Xi'an Polymer Light Technology Corp., Xi'an, China), and oleic acid (OA, 90%; Shanghai Aladdin Biochemical Technology Co., Ltd., Shanghai, China) were used as received. N-Octane, ethyl acetate, and n-hexane were purchased from Shanghai Macklin Biochemical Co., Ltd., Shanghai, China. Amino-acid ligands—alanine, phenylalanine, tryptophan, and cysteine (⩾98%; J&K Scientific, Beijing, China)—were stored in a desiccator prior to use.

Apparatus. Excitation–emission (2D/3D) fluorescence maps and steady-state PL spectra were collected on an Edinburgh Instruments FLS1000 spectrometer equipped with an integrating sphere (Edinburgh Instruments Ltd., Livingston, UK). UV-Vis-NIR absorption spectra were measured on a PerkinElmer Lambda 1050+ spectrophotometer (PerkinElmer, Inc., Waltham, MA, USA). Fourier-transform infrared (FT-IR) spectra were recorded on a Thermo Scientific Nicolet iS50 spectrometer (Thermo Fisher Scientific, Waltham, MA, USA). Powder X-ray diffraction (PXRD) patterns were collected on a PANalytical Empyrean diffractometer (Malvern Panalytical B.V., Almelo, Netherlands); reference patterns were simulated from crystallographic information files (CIFs) using VESTA, and the experimental data were compared with the simulated traces for phase identification. X-ray photoelectron spectroscopy (XPS) was carried out on a Thermo Scientific ESCALAB Xi^+ system (Thermo Fisher Scientific (China) Co., Ltd., Shanghai, China; made in the Czech Republic), and the spectra were processed and peak-fitted using Avantage.

The PLQY reported in this article is in a solution state. It was measured using the Edinburgh Instruments FLS1000 with an integrating sphere by the de Mello absolute method. The sample was placed in a 10 mm path length quartz cuvette with a volume of approximately 3.0 mL, and the solvent was n-hexane. The excitation wavelength was 405 nm with a bandwidth of 2 nm, and the emission was integrated from 420 to

800 nm. Spectra were collected in the order of dark field, blank, and sample. After the software corrected for instrument response and integrating sphere flux, the PLQY was obtained.

Theoretical calculation. The Gaussian 09 program package was used for theoretical calculations in this work. We employed the density functional theory (DFT) method to investigate the molecular geometry and electronic structures, and the polarization function was selected at the B3LYP/6–31+ level. Besides, the molecules' electrostatic potential (ESP) values were calculated by the Multiwfn program package.

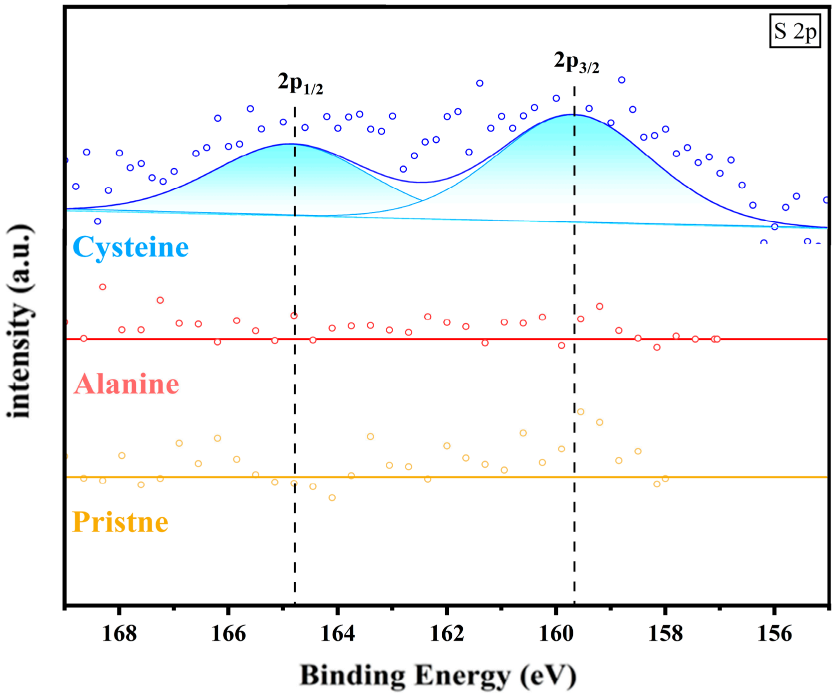

Figure S1. The high-resolution XPS result of S 2p in different modifications.

In the S 2p high-resolution XPS spectrum (Figure S1), only the Cys-QDs produced a definite sulfur signal. In contrast, the S 2p portions of the Ala-QDs and Pri-QDs had essentially no reaction, showing that the sulfur element originated from the side chain of Cys. In the Cys-QDs, there was a pair of classic S 2p spin-orbit splitting peaks, with the primary peak S 2p3/2 positioned at 159.8 eV and S 2p1/2 at 162.8 eV. This is slightly similar to the analysis by Chen et al. on the CsPbI3 system. This peak position can be attributed to the thiolate-like coordination (thiolate-like Pb-S bond)

generated by the mercapto group (-SH) in Cys and the Pb^{2+} on the QDs surface [1]. Furthermore, no free -SH or S-S characteristic peaks in the 163-164 eV range were seen in the spectrogram, showing that the thiol groups of the Cys are predominantly involved in coordination, and only a limited amount may exist in a free form. This result is further confirmed by the removal of the -SH stretching vibration peak in the FTIR. This surface passivation method is consistent with the dual-functional coordination mechanism of Cys with $CsPbI_3$ QDs reported by Chen et al., where the thiol groups achieve defect passivation, and the carboxyl groups coordinate further to stabilize the surface and help improve PLQY and device lifetime. It should be observed that the S $2p_{3/2}$ peak location in the QDs is slightly lower than that in specific publications, which may be attributed to changes in surface electronic states, energy calibration mistakes, or fitting methodologies. However, considering the overall spectral structure and the absence of residual free -SH, it can still be reasonably attributed to Pb-S coordination. Some literature states that peaks associated with Pb-S coordination develop approximately 158 eV after thiol treatment, which further reinforces the reasonableness of the attribution of the S element signal in our experiment [2].

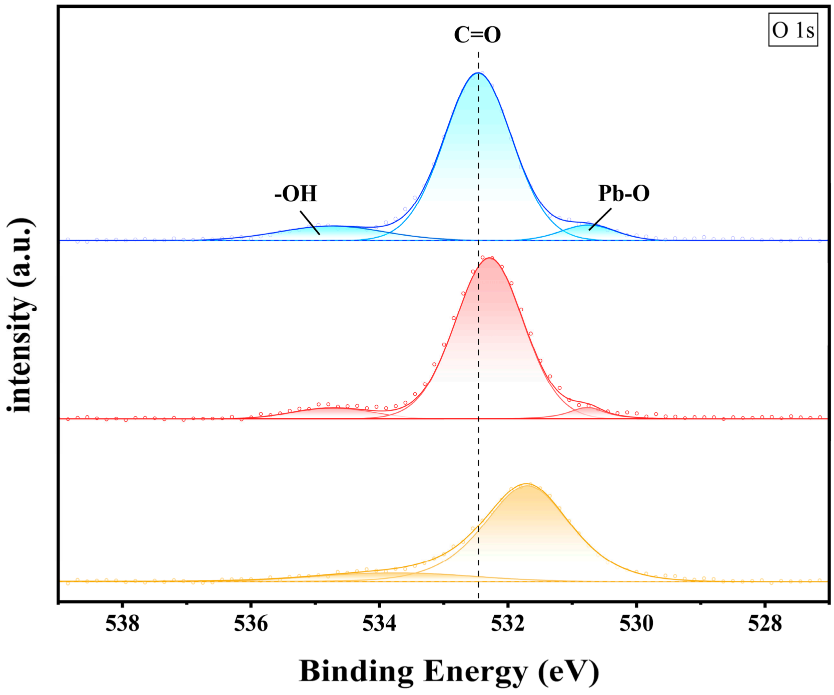

Figure S2. The high-resolution XPS result of O 1s in different modifications.

From the high-resolution XPS spectrum of O 1s (Figure S2), it can be seen that: The central peak of the Pri-QDs is located at 531.6 eV, corresponding to the coordination between the carbonyl oxygen (C=O) of oleic acid/oilamine and Pb2+ [3]; at approxi-mately 534 eV, a weak and broad shoulder peak can be distinguished, which can be attributed to the surface adsorption of -OH/H_2O. After introducing Alanine, the primary peak changes to 532.1 eV, showing a drop in the electron density of the carboxyl oxygen; at the same time, a new shoulder peak arises at 530.7 eV, suggesting that the carboxyl group of Alanine forms an extra Pb-O coordination with Pb^{2+}. Further treatment with Cysteine results in the central peak continuing to shift to 532.5 eV, and the shoulder peak at 530.7 eV becomes significantly enhanced, indicating an increase in the number of Pb-O coordination, and the surface defect passivation effect is better than that of the Ala-QDs and Pri-QDs [4].

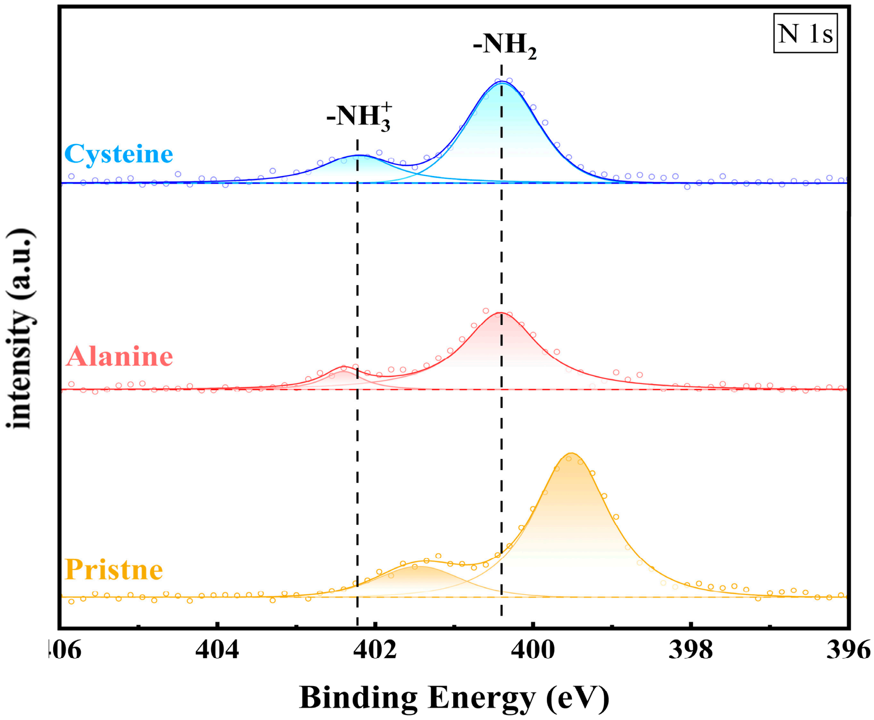

Figure S3. The high-resolution XPS result of N 1s in different modifications.

In the N 1s high-resolution XPS spectrum (Figure S3), all three QDs reveal a double-peak shape. Compared to the central peak at 399.6 eV in the Pri-QDs, the amino acid-modified QDs show a further shift to around 400.4 eV. This peak is attributable to the nitrogen atom in the =N–C–NH_2 link of the FA+ cation within the perovskite lattice. The insertion of amino acid ligands induces a modest shift in the electrical environment of the nitrogen in FA+. Another high-binding-energy peak changed from 401.4 to 402.4 eV, attributable to the NH3+ group in the amino acid molecule, which exists in a coordinated or hydrogen-bonded state [1]. This change trend might be read as amino acids competitively interacting with Pb2+ during crystal nucleation or growth, undermining the stable embedding of FA+. This prevents some organic cations from successfully entering the lattice, reducing the surrounding charge density and somewhat raising the binding energy. Although the order of FA+ is slightly altered, the coordination generated by amino acids effectively passivates surface imperfections, hence increasing luminous performance [5].

Table S1. Chemical reagents, molecular formulas, and cost details for ligand and additive screening in perovskite QD and LED passivation.

**Methods**	**Molecular formula**	**MW (g/mol)**	**Purity**	**Price ($/g)**	**Experimental dosage (g, once)**	**Once cost ($)***	**Source**
n-hexadecylphosphocholine	$C_{21}H_{46}NO_4P$	407.57	⩾97%	431.40$/g	0.0421g	18.162$	[6]
SB3-12	$C_{17}H_{37}NO_3S$	335.55	⩾97%	46.26$/g	0.0346g	1.601$	[7]
3-(N,N-Dimethylpalmitylammonio)propanesulfonate	$C_{21}H_{45}NO_3S$	391.65	⩾98%	50.22$/g	0.040g	2.007$	[8]
Poly(carboxybetaine acrylamide)	$(C_{11}H_{20}N_2O_3)_x$	228.29	⩾99%	895.00$/g	0.0230g	20.612$	[9]
DCzGPC	$C_{38}H_{42}N_3O_8P$	722.26	⩾99%	303.79$/g	0.0729g	22.155$	[10]
**L-Alanine**	$\mathbf{C_3H_7NO_2}$	**89.09**	**⩾99%**	**15.60$/g**	**0.0090g**	**0.140$**	**This Work**
**L-Phenylalanine**	$\mathbf{C_9H_{11}NO_2}$	**165.19**	**⩾98%**	**14.76$/g**	**0.0169g**	**0.249$**	
**L-Tryptophan**	$\mathbf{C_{11}H_{12}N_2O_2}$	**204.23**	**⩾99%**	**19.36$/g**	**0.0206g**	**0.399$**	
**L-Cysteine**	$\mathbf{C_3H_7NO_2S}$	**121.16**	**⩾98.5%**	**6.74$/g**	**0.0123g**	**0.083$**	

*The prices of the above-mentioned raw materials are sourced from the Sigma-Aldrich official website (https://www.sigmaaldrich.cn/CN/zh) and the Polymer Source official website (https://www.polymersource.ca/), as well as estimated based on literature.

Ref.

(1) Chen, D.; Ko, P. K.; Li, C.-H. A.; Zou, B.; Geng, P.; Guo, L.; Halpert, J. E. 24. *ACS Energy Lett.* **2023**, *8* (1), 410–416. https://doi.org/10.1021/acsenergylett.2c02243.

(2) Fiuza-Maneiro, N.; Sun, K.; López-Fernández, I.; Gómez-Graña, S.; Müller-Buschbaum, P.; Polavarapu, L. 6. *ACS Energy Lett.* **2023**, *8* (2), 1152–1191. https://doi.org/10.1021/acsenergylett.2c02363.

(3) Zhang, J.; Zhang, D.; Zhou, X.; Lian, L.; Shen, C.; Su, C.; Fang, S.; Liang, X.; Yuan, F.; Hou, L.; Yuan, Y.-X. 2. *Nano Lett.* **2024**, *24* (39), 12196–12203. https://doi.org/10.1021/acs.nanolett.4c03229.

(4) Xu, J.; Chen, H.; Grater, L.; Liu, C.; Yang, Y.; Teale, S.; Maxwell, A.; Mahesh, S.; Wan, H.; Chang, Y.; Chen, B.; Rehl, B.; Park, S. M.; Kanatzidis, M. G.; Sargent, E. H. 32. *Nat. Mater.* **2023**, *22* (12), 1507–1514. https://doi.org/10.1038/s41563-023-01705-y.

(5) Vásquez-Montoya, M.; Montoya, J. F.; Ramirez, D.; Jaramillo, F. 26. *Journal of Energy Chemistry* **2021**, *57*, 386–391. https://doi.org/10.1016/j.jechem.2020.08.059.

(6) Morad, V.; Stelmakh, A.; Svyrydenko, M.; Feld, L. G.; Boehme, S. C.; Aebli, M.; Affolter, J.; Kaul, C. J.; Schrenker, N. J.; Bals, S.; Sahin, Y.; Dirin, D. N.; Cherniukh, I.; Raino, G.; Baumketner, A.; Kovalenko, M. V. 57. *Nature* **2024**, *626* (7999), 542–548. https://doi.org/10.1038/s41586-023-06932-6.

(7) Lee, A.; Kim, J.; Lee, D.; Song, M. H. 55. *ACS Appl. Electron. Mater.* **2023**, *5* (10), 5325–5331. https://doi.org/10.1021/acsaelm.2c01781.

(8) Jing, Y.; Low, A. K. Y.; Liu, Y.; Feng, M.; Lim, J. W. M.; Loh, S. M.; Rehman, Q.; Blundel, S. A.; Mathews, N.; Hippalgaonkar, K.; Sum, T. C.; Bruno, A.; Mhaisalkar, S. G. 3. *Advanced Materials* **2024**, *36* (44), 2405973. https://doi.org/10.1002/adma.202405973.

(9) Jin, H.; Yeong Park, G.; Kyong Kim, M.; Cha, J.; Seok Ham, D.; Kim, M. 4. *Chemical Engineering Journal* **2023**, *459*, 141531. https://doi.org/10.1016/j.cej.2023.141531.

(10) Kirsch, C.; Naujoks, T.; Haizmann, P.; Frech, P.; Peisert, H.; Chassé, T.; Brütting, W.; Scheele, M. 5. *ACS Appl. Mater. Interfaces* **2023**, *15* (27), 32744–32752. https://doi.org/10.1021/acsami.3c05756.
